# Allele frequency deviation (AFD) as a new prognostic model to predict overall survival in lung adenocarcinoma (LUAD)

**DOI:** 10.1186/s12935-021-02127-z

**Published:** 2021-08-26

**Authors:** Aisha Al-Dherasi, Yuwei Liao, Sultan Al-Mosaib, Rulin Hua, Yichen Wang, Ying Yu, Yu Zhang, Xuehong Zhang, Raeda Jalayta, Haithm Mousa, Abdullah Al-Danakh, Fawze Alnadari, Marwan Almoiliqy, Salem Baldi, Leming Shi, Dekang Lv, Zhiguang Li, Quentin Liu

**Affiliations:** 1 Center of Genome and Personalized Medicine, Institute of Cancer Stem Cell, Dalian Medical University, Dalian, 116044 Liaoning People’s Republic of China; 2 Department of Biochemistry, Faculty of Science, Ibb University, Ibb, Yemen; 3 Yangjiang Key Laboratory of Respiratory Diseases, Yangjiang Peoples Hospital, Yangjiang, Guangdong People’s Republic of China; 4 Department of Computer Science and Technology, Sahyadri Science Collage, Kuvempu University, Shimoga district, Karnataka India; 5 State Key Laboratory of Genetic Engineering, School of Life Sciences and Human Phenome Institute, Fudan University, 2005 Songhu Road, Shanghai, 200438 People’s Republic of China; 6 Department of Clinical Biochemistry, College of Laboratory Diagnostic Medicine, Dalian Medical University, Dalian, 116044 Liaoning People’s Republic of China; 7 Department of Urology, First Affiliated Hospital of Dalian Medical University, Dalian Medical University, Dalian, 116044 Liaoning People’s Republic of China; 8 Department of Food Science and Engineering, College of Food Science and Technology, Nanjing Agricultural University, Nanjing, 210095 Jiangsu People’s Republic of China; 9 Key Lab of Aromatic Plant Resources Exploitation and Utilization in Sichuan Higher Education, Yibin University, Yibin, 644000 Sichuan China

**Keywords:** Allele frequency deviation (AFD), Lung Adenocarcinoma (LUAD), Overall survival

## Abstract

**Background:**

Lung adenocarcinoma (LUAD) remains one of the world’s most known aggressive malignancies with a high mortality rate. Molecular biological analysis and bioinformatics are of great importance as they have recently occupied a large area in the studies related to the identification of various biomarkers to predict survival for LUAD patients. In our study, we attempted to identify a new prognostic model by developing a new algorithm to calculate the allele frequency deviation (AFD), which in turn may assist in the early diagnosis and prediction of clinical outcomes in LUAD.

**Method:**

First, a new algorithm was developed to calculate AFD using the whole-exome sequencing (WES) dataset. Then, AFD was measured for 102 patients, and the predictive power of AFD was assessed using Kaplan–Meier analysis, receiver operating characteristic (ROC) curves, and area under the curve (AUC). Finally, multivariable cox regression analyses were conducted to evaluate the independence of AFD as an independent prognostic tool.

**Result:**

The Kaplan–Meier analysis showed that AFD effectively segregated patients with LUAD into high-AFD-value and low-AFD-value risk groups (hazard ratio HR = 1.125, 95% confidence interval CI 1.001–1.26, p = 0.04) in the training group. Moreover, the overall survival (OS) of patients who belong to the high-AFD-value group was significantly shorter than that of patients who belong to the low-AFD-value group with 42.8% higher risk and 10% lower risk of death for both groups respectively (HR for death = 1.10; 95% CI 1.01–1.2, p = 0.03) in the training group. Similar results were obtained in the validation group (HR = 4.62, 95% CI 1.22–17.4, p = 0.02) with 41.6%, and 5.5% risk of death for patients who belong to the high and low-AFD-value groups respectively. Univariate and multivariable cox regression analyses demonstrated that AFD is an independent prognostic model for patients with LUAD. The AUC for 5-year survival were 0.712 and 0.86 in the training and validation groups, respectively.

**Conclusion:**

AFD was identified as a new independent prognostic model that could provide a prognostic tool for physicians and contribute to treatment decisions.

**Supplementary Information:**

The online version contains supplementary material available at 10.1186/s12935-021-02127-z.

## Background

Lung cancer is the most common cause of cancer incidence and death-causing conditions in China and the world [1, 2]. Non-small cell lung cancer (NSCLC) accounts for nearly 80% of lung cancer, and it is histopathologically classified into two main subtypes: lung squamous cell carcinoma (LUSC) and lung adenocarcinoma (LUAD) [3], where the latter is the most common type, with a survival rate of approximately 15% within 5 years [4, 5]. These histological subtypes play the main role of determining the therapeutic options. Although patients with NSCLC receive different treatments, whether early-stage surgical treatment or other potential curative treatments for different stages, the prognosis of patients with NSCLC in the early stages remains poor, with a relapse rate of approximately 40% in patients within 5 years [6] and a survival rate of 50–60% [7, 8]. These information indicate the existence of some individual cases of high-risk among patients who are in the early stages of the disease. Therefore, patients need to be diagnosed in the early stages, and a reliable prognostic biomarker or prognostic factors to identify high-risk individuals are urgent and considerably important for NSCLC.

There is a range of different and varied studies in their results conducted at the recent time to identify the prognostic factors and/or prognostic biomarkers for the diagnosis of patients with lung adenocarcinoma (LUAD). These biomarkers may include one of the following types: (1) biomarkers associated with the risk of development of toxicity related to certain medications in patients and this biomarker is single nucleotide polymorphism (SNP) haplotype; (2) Biomarkers indicating the recurrence of the disease after surgical removal, they are found on the tumor or secreted by the tumor such as some proteins; (3) The presence of genetic mutations targeted by the therapy or the level of gene expression, both of which act as biomarkers; (4) Finally, the number of cancer cells circulating or the tumor metabolic activity may be another vital indicator. Many studies have demonstrated tumor mutation burden (TMB) as a biomarker for patients with LUAD [9]. For example, Rizvi et al. [10] demonstrated that high TMB levels were correlated with improved ORR and prolonged PFS in a retrospective analysis of patients with NSCLC. Talvitie et al. [11] in its study on lung adenocarcinoma patients has shown that TMB is an independent biomarker for predicting survival, as patients with TMB greater than or equal to 14 mutations/MB had a longer survival than patients with TMB less than 14 mutations/MB. In another study, Jiao et al. [12] proved that TMB was a negative biomarker to predict survival for LUAD patients, where the TMB was low in the group of patients with EGFR-mutation. In addition, change in mean variant allele frequencies (dVAF) has been identified as a predictor of clinical outcomes in NSCLC and UC [13]. Allele frequency deviation (AFD) refers to the degree of deviation between the single nucleotide variant (SNV) allele frequency to tumor samples and that of matched control samples, it can reflect the disease stats of patients, as demonstrated in another study on AFD involving patients with cervical cancer revealed that AFD was positively correlated with therapy response and it helped in estimating progression-free survival [14].

On the basis of the previous studies on many different prognostic biomarkers, particularly the AFD-related study [14], the relationship between AFD and overall survival was identified in patients with LUAD in the current study by developing a new algorithm for measuring AFD and then evaluating its predictive performance to predict the survival of LUAD patients in the early stages as an independent prognostic model. This study is considered the first study to report the direct association of AFD for the prediction of patients survival, which may contribute and help in the early detection of LUAD patients and making effective clinical decisions regarding potential individual treatment.

## Materials and methods

### Data source

The raw data of whole-exome sequencing (WES) with clinical information related to patients with lung adenocarcinoma were obtained from Fudan University. The total number of patients after excluding those with insufficient clinical information was 102. They were randomly divided into two groups: training group, which included 54 patients, and validation group, which included 48 patients. The basic clinical characteristics included in the analysis are as follows: history of smoking, pT stage, age, sex, and tumor size. The details are provided in (Table 1). The data analysis process was carried out on the data collected by Fudan University that was previously used in another study [15] which was conducted according to the ethical standards (Fudan University Shanghai Cancer Center Institutional Review Board No. 090977-1). Informed consents of patients or their relatives were obtained while donating a samples to the tissue bank of Fudan University Shanghai Cancer Center [15]. For more information pertaining to the data analyzed in our study, the data can be accessed and obtain from the European Genome-phenome Archive (EGA) via using the following access code: EGAS00001004006.

**Table 1:** Baseline Characteristics at Diagnosis

Characteristic	(N = 102)	Characteristic	(N = 102)
Age—yr (no.)		T1a	
Median	61.5	T1b	40
Mean	61.8	T2a	27
Range	37–84	T2b/T4	33
Age category—no. (%)		Sex—no. (%)	
≤ 60 yr	44 (43%)	Male	49 (48%)
> 60 yr	58 (57%)	Female	53 (52%)
Smoking status—no. (%)		Tumor_size	
Former/current	31 (30%)	Mean	2.4
Never	71 (70%)	Median	2
pT(no.)		Range	0.7–6

### Alignment and quality control

**Fig. 1:**
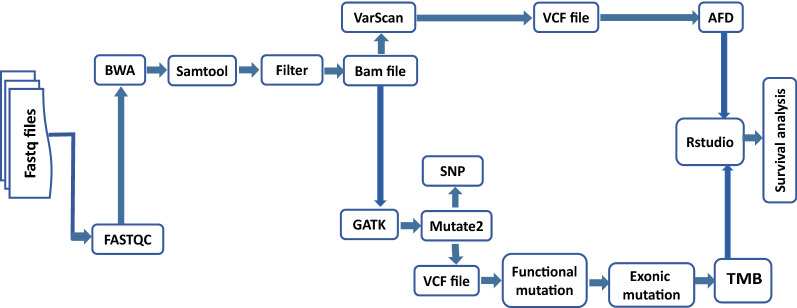
Whole exome sequencing analysis flowchart

In-house pipelines were used to process the sequencing of 102 WES data. Tumor and normal sample quality data were evaluated using FastQC (http:/www.bioinformatics.babraham.ac.uk/projects/fastqc/), including sequence length distribution, GC content, aspect of per-base quality, sequence duplicate levels, kemer content, and over-represented sequences [14]. Sequencing readings were aligned with the human reference genome (hg38) by using the Burrows-Wheeler Aligner (BWA) software package with default parameters [16]. The reads that were mapped in multiple genome positions were removed. Then, the quality of the map was accessed using SAM tools flagset [17]. All the genome sites for somatic variants were called by using VarScan2 [18] software with parameters of base quality higher than 30 and supporting reads ≥ 200 (Fig. 1).

### Calling of SNV from WES

After all the readings were mapped to the human reference genome (hg38) by using BWA [16], Picard 1.67 was used to mark the duplicate readings realigned around the known indels. Base quality recalibration was performed using GATK version 3.7 [19]. Somatic mutations were called using Mutect2 after insuring that the following criteria have been met: first, the difference of mutant allele fraction (MAF) between the tumor and normal sample in the same patient was more than one percentage; second, in both tumor and normal samples, the sequencing coverage was more than 200; third, the alternative readings in the tumor samples were more than 10; and fourth, the corrected p-value was less than 0.05. SNVs were annotated using ANNOVAR in multiple databases [20] and further filtered with population frequency in ExAC, 1000 Genomes, dbSNP138.

### Allele frequency deviation (AFD)

**Fig. 2:**
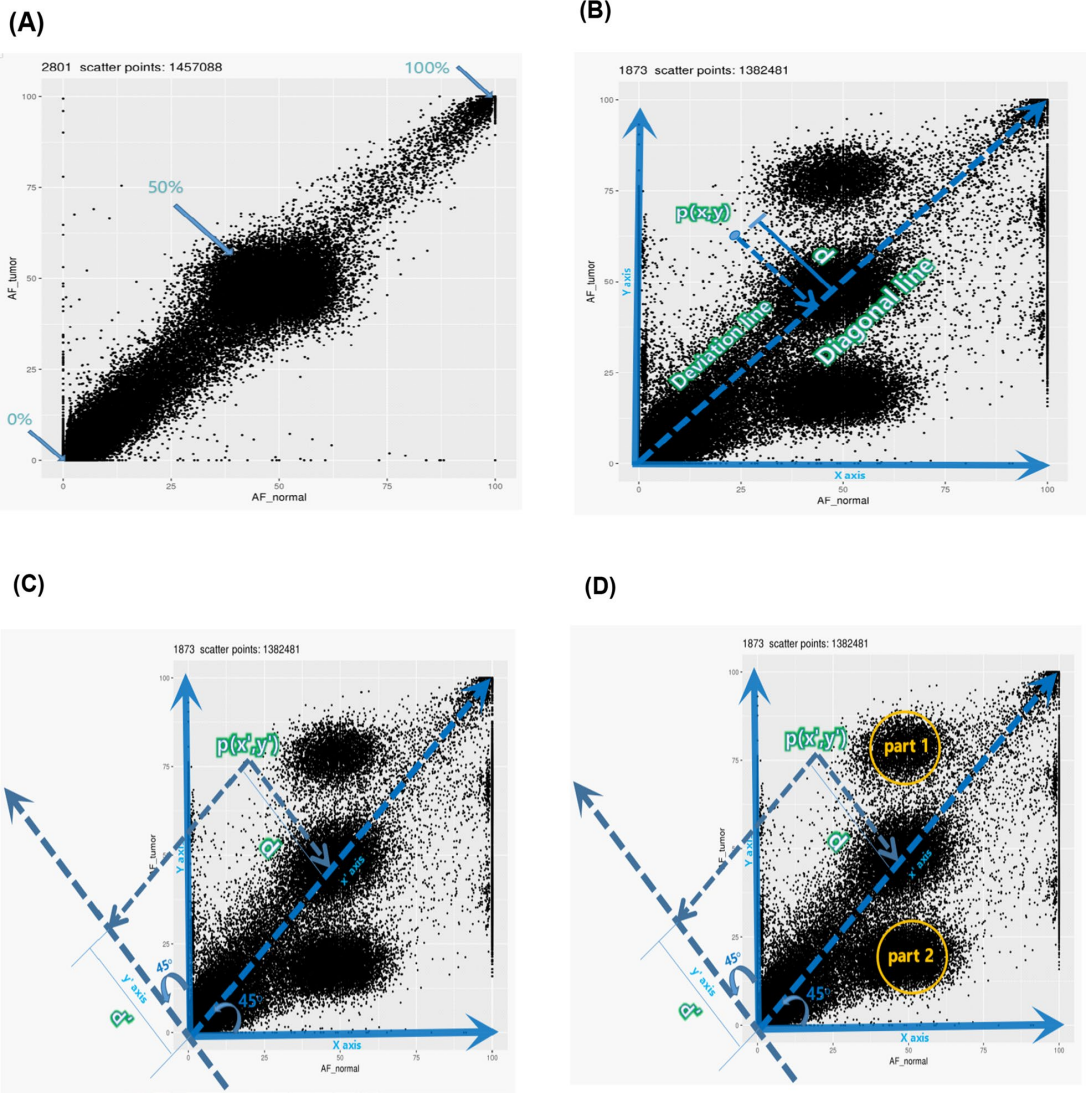
Calculation of Allele Frequency Deviation. **A** Qualified distribution for every sites of Variant allele frequency (VAF) in normal cells should be lies around wild type (0%), heterozygous (50%) and homozygous (100%). **B** Diagonal line on each point that have the same VAF in both tumor and normal samples. **C**, **D** Transporation of X and Y coordinates by −45°

Variant allele frequency (VAF) of exome sites for 102 samples were called by using VarScan2 [18] software with the base quality higher than 30 and read depth ≥ 200, the WBC sample was used as a control to calibrate possible errors of the sequence and germline variants during the calculation of the VAF (Fig. 1). Then variant allele frequencies were used to calculate AFD for each patient. As displayed in (Fig. 2), a scatter plot was first created for all the detected genomic sites of the patient, with Y axis representing the VAF of a tumor sample and X axis representing the VAF of a paired normal sample. Second, a diagonal line, on which the points have the same VAF between both samples, was created. The distance from each point to this diagonal line was calculated and defined as *d*_*i*_ of the *i−*th point. Third, the X,Y coordinates were transposed by − 45°; thus, *d*_*i*_ is equal to the absolute value of the Y axis of *i* point and could be calculated using the Eq. (1):

1 $$di = \left| {yi^{^{\prime}} } \right| = \left| {xi*\sin \left( { - \frac{{\uppi }}{4}} \right) + yi*{\text{cos}}\left( { - \frac{{\uppi }}{4}} \right)} \right|$$

where *y*_*i*_*’* is the transposed Y-axis value of the i point, the *x*_*i*_, *y*_*i*_ is the original X and Y axis values. Finally, the AFD of the patients was calculated as in the Eq. (2):

2 $$AFD = \frac{{\mathop \sum \nolimits_{i = 1}^{n} \left( {di} \right)}}{{\text{n}}}$$

where *di* represent the distance value of all points i that are deviated from the diagonal line, n represent the total number of point.

### Tumor mutation burden (TMB)

In short, the tumor mutation burden (TMB) is defined as the total number of somatic (nonsynonymous) mutations, which include the small insertions and deletions (INDELs) and single nucleotide variants (SNVs) for each megabase [21, 22]. The golden standard method of measuring the TMB is through the use of WES, which can detect somatic mutations in the entire exome and thus give a comprehensive perception of all mutations that can contribute to the progress of the tumor at level of cost that is considered lower than the WGS [23]. The Quantile method based on TMB measurements was used to determine the appropriate cutting values [24].

### Statistical analysis

Spearman correlation test was conducted to determine the correlation between factors, such as AFD and TMB. Kaplan–Meier (K-M) analysis was used to evaluate the differences in patient survival time between the high- and low-AFD value groups of patients with LUAD. The P values and HR (95% confidence interval [CI]) were determined via log-rank test and univariate Cox regression analysis to detect the significant differences between the groups. Multivariable Cox regression analysis performed to evaluate AFD independence. The ROC curve was used to estimate the performance of AFD by comparing the AUC. Statistical significance was identified as P ≤ 0.05. All statistical analyses were performed using version 3.5.1 of the R language.

## Results

### Patients characteristic

The main histological subtype in this study was lung adenocarcinoma (LUAD). The range of the patient’s age was between 37 and 84 years (61.5 years as a median age). Fifty-three (52%) patients were female and 49 (48%) were male; their output status was zero or one; 70% of the patients never smoked, while 30% were former/current smokers. Forty (39.2%) had stage T1a, twenty-seven patients (26.4%) had stage T1b, thirty-three patients (32.3%) had stage T2a, one patient had stage T2b (98%) and one patient had stage T4 (98%) (Table 1) (Additional file 1: Table S1). The patients have not received any neoadjuvant treatment.

### Relationship between AFD and TMB

**Fig. 3:**
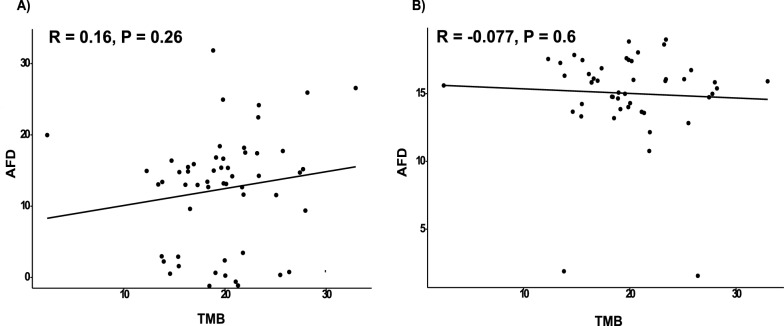
Spearman Correlation between the AFD and TMB. The association between AFD and TMB in patients with LUAD in the training group (**A**) and validation group (**B**)

In order to find out if the AFD and TMB are related, we performed a Spearman correlation test. Figure 3(A) shows the correlations between AFD and TMB in patients with LUAD. Spearman correlation coefficient showed that the p-value of the test was more than the significance level of 0.05. Therefore, AFD and TMB were not significantly associated at a correlation coefficient of 0.16 and p-value of 0.26 for the training group. In the validation group, the result also showed no correlation between AFD and TMB, with a p-value of 0.6 and correlation coefficient = −0.077 (Fig. 3B).

### Allele frequency deviation shows an active power to predict patient outcomes

**Fig. 4:**
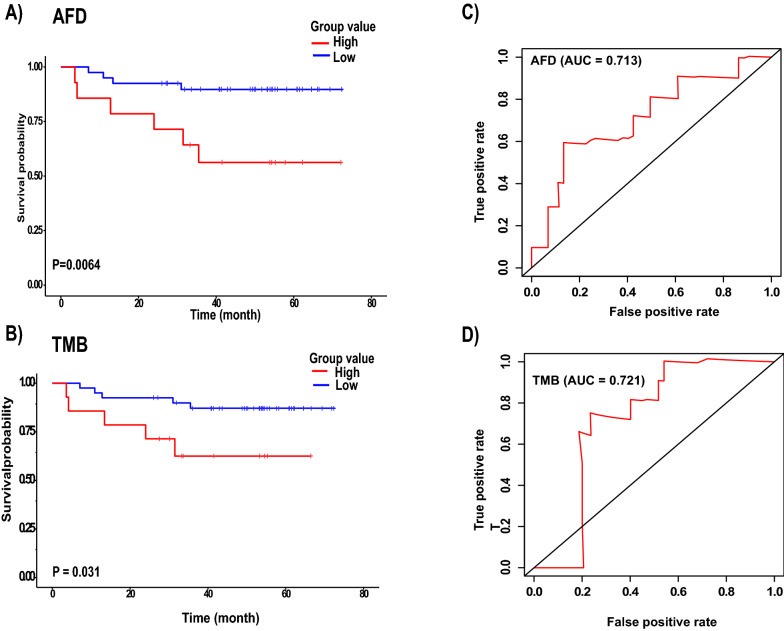
Performance of AFD and TMB in the training group. **A** & **B** Kaplan–Meier survival curve analysis of AFD and TMB respectively. **C** & **D** The receiver operating characteristic (ROC) curve for the 5-year survival of patients with LUAD in AFD and TMB respectively

**Fig. 5:**
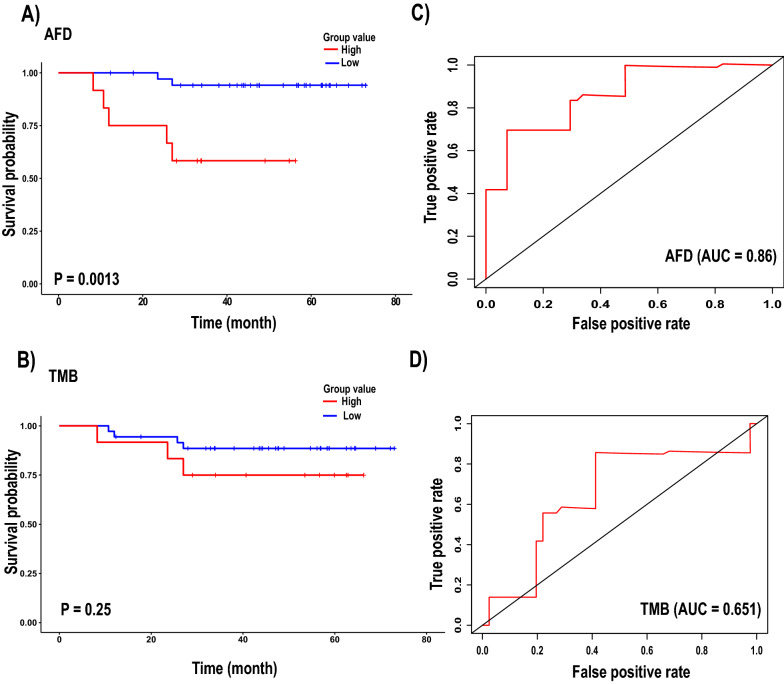
Performance of AFD and TMB in the validation group. **A** & **B** Kaplan–Meier survival curve analysis of AFD and TMB respectively. **C** & **D** The receiver operating characteristic (ROC) curve for the 5-year survival of patients with LUAD in AFD and TMB respectively

A time-dependent curve was used to evaluate the sensitivity and specificity of AFD and TMB for OS prediction in the training and validation groups. The AFD and TMB significantly achieved almost the same AUC values of 0.713 and 0.721 (Fig. 4C and D), respectively, in the training group, while in the validation group, AFD achieved an AUC of 0.86 and TMB achieved 0.65 (Fig. 5C and D). These results demonstrated that AFD has the good power and efficient prognostic performance to predict the survival of patients with LUAD, which is reflected by the AUC value.

### Overall survival

Considering that TMB and AFD are continuous variables and the cutting points for these variables are still not uniformly established, therefore in our study, we assumed that the risk of death is associated with the rise of AFD values, and in order to select a group of patients with high AFD values as a high-risk group and separate them from the low AFD values group as a low-risk group, we used the quantile method to get the correct cutting point based on AFD values. In the training set, the mean value of AFD was 13.74 (0.15–33.18), while it was 19.81 (2.5–32.97) for TMB. The AFD cutoff points at 75% quantile were 17.93 and 22.028 mutation/Mb for the AFD and TMB in the training set, respectively, and 16.7 and 23.2 mutation/Mb for the AFD and TMB in the validation set, respectively, thus dividing the patients into high and low-value groups. The Kaplan–Meier curve estimated the OS at 31 months as 89.7% (95% confidence interval [CI] 80.6–99.8) in the low-AFD-value group and 64.3% (95% CI 43.5–95) in the high-AFD-value group (Table 2). A gradual decrease was observed in survival from 78.6% at 12 months to 52.2% at 35 months in the high-AFD-value group. In the training group, the OS of patients who belong to the low-AFD-value (low-risk) group was significantly longer than that of patients who belong to the high-AFD-value (high-risk) group, with 10% lower risk of death and 42.8% higher risk of death for both groups, respectively (HR for death = 1.10; 95% CI 1.01–1.2, p = 0.03) (Tables 2 and 4). The patients in the high and low-AFD-value groups included in the survival analysis according to their cutoff points were 14 and 40, respectively. In the validation group, OS was found to be significantly longer in the low-AFD-value (low-risk) group than in the high-AFD-value (high-risk) group, with 5.5% lower risk of death and 41.6% higher risk of death for both groups, respectively (HR = 3.1, 95% CI 1.4–6.60, p = 0.003) (Tables 3 and 4). The patients in the high and low-AFD-value groups included in the survival analysis according to their cutoff points were 12 and 36, respectively.

**Table 2:** Overall survival in AFD, TMB and Kaplan–Meier estimates in the training group

Variable	Low-value group (n = 40)	High-value group (n = 14)
AFD		
Deaths—no. (%)^a^	4	6
Data censored^b^	36	8
Median overall survival—mo (95% CI)	NE	NE
The overall survival (95% CI) by Kaplan–Meier estimation		
12 mo	95% (88.5–100)	78.6% (59.8–100)
31 mo	89.7% (80.6–99.8)	64.3% (43.5–95)
35 mo	NA	56.2% (35.2–90)
TMB		
Deaths—no. (%)^a^	5	5
Data censored^b^	35	9
Median overall survival—mo (95% CI)	NE	NE
The overall survival (95% CI) by Kaplan–Meier estimation		
13 mo	92.5(84.7–100)	78.6 (59.8–100)
31 mo	89.9 (80.9–99.8)	62.5 (41–95.3)
35 mo	87.1 (77.2–98.3)	NA

^a^Represent the hazard ratio for death

^b^Indicate the date for censorship of patients on the date the patient was last known to be alive

NA indicate that there is no available events

NE represent that the value could not be estimated

**Table 3:** Overall survival in AFD, TMB and Kaplan–Meier estimates in the validation group

Variable	Low-value group (n = 36)	High-value group (n = 12)
AFD		
Deaths—no. (%)^a^	2	5
Data censored^b^	34	7
Median overall survival—mo (95% CI)	NE	NE
The overall survival (95% CI) by Kaplan–Meier estimation		
23 mo	97.1% (91.5–100)	NA
25 mo	NA	66.7% (44.7–99.5)
27 mo	94.1% (86.5–100)	58.3% (36.2–94.1)
TMB		
Deaths—no. (%)^a^	4	3
Data censored^b^	32	9
Median overall survival—mo (95% CI)	NE	NE
The overall survival (95% CI) by Kaplan–Meier estimation		
10 mo	97.2 (92.0–100)	NA
8 mo	NA	91.7 (77.3–100)
27 mo	88.5 (78.6–99.8)	75.0 (54.1–100)

^a^Represent the hazard ratio for death

^b^Indicate the date for censorship of patients on the date the patient was last known to be alive

NA indicate that there is no available events

NE represent that the value could not be estimated

**Table 4:** Univariate and multivariate cox regression analysis of AFD, TMB and overall survival in patients with LUAD

Variables		Patients (N)	Univariate analysis		Multivariate analysis	
			HR (95% CI)	P	HR (95% CI)	P
Training group						
Sex	Female/Male	27/27	1.581 (0.44–5.61)	0.47	0.33 (0.025–4.49)	0.40
Age	≤ 60/ > 60	24/30	1.03 (0.96–1.10)	0.31	0.99 (0.91–1.079)	0.89
Tumor_size	≤ 2/ > 2	28/27	1.279 (0.81–2.00)	0.28	1.2 (0.677–2.395)	0.45
Smoking	N/(F/C)	36/18	0.467 (0.13–1.61)	0.22	0.20 (0.017–2.45)	0.21
pT	T1/ T2	35/19	1.297 (0.366–4.6)	0.68	0.63 (0.104—3.7)	0.61
TMB	< 22/ ≥ 22	40/14	1.088 ( 0.96–1.23)	0.17	1.064 (0.90–1.25)	0.44
AFD	< 17.9 / ≥ 17.9	40/14	1.100 (1.008–1.2)	**0.03**	1.125 (1.001–1.26)	**0.04**
Validation group						
Sex	Female/Male	26/22	0.17 (0.021–1.48)	0.11	0.013 (0.003–0.46)	0.017
Age	≤ 60/ > 60	19/29	0.97 (0.91–1.04)	0.48	0.975 (0.87–1.089)	0.65
Tumor_size	≤ 2/ > 2	23/25	2.5 (1.15–5.54)	0.02	1.3 (0.25–6.61)	0.75
Smoking	N/(F/C)	35/13	0.87 (0.17–4.52)	0.87	0.06 (0.002–2.15)	0.12
pT	T1/ T2/T4	32/15/1	1.52 (0.63–3.83)	0.37	3.5 (0.094–0.99)	0.49
AFD	< 16.76/ ≥ 16.76	36/12	3.1 (1.4–6.60)	**0.003**	4.62 (1.22–17.4)	**0.02**

Bold values indicate the significant values < 0.05

CI, confidence interval; C, current; F, Former; HR, hazard ratio; N, Never

The one-sided stratified log-rank p-values were 0.0064 (Fig. 4A) and 0.0013 (Fig. 5A) for the training and validation groups, respectively, indicating a significant difference between the two groups regardless of the number of patients in each group. The result also showed that patients with high AFD values were at higher risk of death than patients with low AFD values. The Kaplan–Meier curve for TMB in the training group showed that the high-level patients had significantly shorter OS than the low-level patients, with 35.7% higher risk of death (HR = 1.08, 95% CI 0.96–1.2, p = 0.17). Thus, the OS was 62.5% at 31 months (95% CI 41–95.3) in the high-level TMB group and 89.9% (95% CI 80.9–99.8) in the low-level TMB group (Tables 2 and 4). The number of patients in the high-level group was 40, while it was 14 in the low-level group. The one-sided stratified log-rank p-value was notably 0.03, indicating the difference between the two groups in regard to OS (Fig. 4B). In the validation group, no significant differences were found between the two groups in the Kaplan–Meier curve (Fig. 5B). The numbers of patients in the high and low-level groups were 36 and 12, respectively.

### AFD as an independent prognostic factor

Herein, univariate and multivariable Cox regression analyses were conducted in the training and validation groups to assess the contribution of AFD as an independent prognostic factor for patients with LUAD. AFD and other clinicopathological factors, including gender, smoking, age, pT, and tumor-size, were used as covariates. Univariate regression analysis indicated that AFD (p = 0.03) was significantly associated with patient survival, while sex (p = 0.47), age (p = 0.31), tumor size (p = 0.28), smoking (p = 0.22), pT (P** = **0.68) and TMB (p = 0.17) were not significantly associated with patient survival in the training group, as shown in (Table 4). For the validation group, the analysis showed that AFD (p = 0.003) was the only factor correlated with patient survival; the other clinical factors did not show any association with patient survival (Table 4). The corresponding multivariable cox regression analysis confirmed that the AFD in the training (HR = 1.125, 95% CI = 1.001–1.26, P = 0.04) and validation (HR = 4.62, 95% CI 1.22–17.4, P = 0.02) groups was an independent prognostic factor (Table 4). These results showed that AFD is an independent risk factor that could be used as a prognostic tool for patients with LUAD to assist in the early diagnosis for LUAD patients.

## Discussion

The time of survival differs due to the different stages of LUAD among patients, as this type of cancer is heterogeneous. Many clinical variables have taken up a wide area in the field of predicting the diagnosis and treatment of patients with LUAD, but the results are uneven. The most important factors are TNM stage, race, age, tumor size, and gender these are factors related to the patient. Other factors related to the tumor also contribute to the prediction of the outcomes and treatment of patients, including the invasion of blood vessels and cell differentiation [25–29].

In the current study, the patients with high AFD values were assumed to be at a high risk compared with those with low AFD values. Therefore, AFD may act as an indicator of the progress of the disease and the survival rate of patients. For confirmation, the patients were divided into two groups. The first group consisted of patients with high AFD values, while the second group consisted of those with low AFD values. The quantile method was used to obtain the appropriate cutoff point to separate patients into two groups in a scientific and unbiased manner. Through this cutoff value, a significant difference was obtained between the high and low-risk groups. Thus, AFD had a clear effect in predicting the survival of patients and identifying patients who are at high risk. Multivariable cox regression analysis showed that AFD is an independent prognostic tool capable of predicting survival in patients with LUAD. In addition, ROC analysis showed that AFD has the effect power to predict overall survival of patients.

Previous studies have shown that TMB was significantly correlated with immune checkpoint inhibitors (ICIs), such as PD-L1 and PD-1, and other biomarkers, including EGFR and TP53 [30–32]. In the present research, the relationship between AFD and TMB were evaluated, and the results showed no correlation between the two. Furthermore, the AUC of the prediction for patient survival in AFD and TMB was high and almost the same, suggesting that AFD had a substantial efficiency not less than the efficiency of TMB to predict overall survival. In addition, these results are consistent with the findings in the Kaplan–Meier analysis for patients with LUAD, with a high statistical significance of AFD in the prediction. The patients were also divided by AFD into high and low-value risk group, the patients with high AFD value had shorter OS than those with low AFD value. On the contrary, univariate and multivariable cox regression analyses showed that TMB tended to be a non-independent prognostic factor for predicting the survival of patients with LUAD, and no significant association was observed between TMB and LUAD patients survival. This finding is consistent with that of previous studies [33, 34], which showed that TMB was significantly related to the prediction of the response of patients to the medications used in order to determine their effectiveness. Interestingly, AFD displayed a efficiency and predictive ability in both analyses and emerged as an independent prognostic factor.

A number of studies have reported that tumor size is a prognostic factor used to predict patient progression and outcomes [35]. A previous study related to AFD demonstrated the effectiveness of AFD in predicting the benefit and response of patients with cervical cancer to treatment, and the predicted evidence of metastases was better than that of tumor size [14]. In the present study, AFD was shown to be independent of tumor size, and patients with high AFD values had worse prognosis than patients with low AFD values. Therefore, AFD can be considered as a prognostic factor for predicting the outcome of patients with LUAD, consequently suggesting the use of AFD in clinical application for the purpose of early diagnosis of lung adenocarcinoma patients.

AFD is still a new model that has not yet been used as a prognostic model for the prediction of clinical outcomes in lung adenocarcinoma or any other type of cancer. Therefore, this study is the first to show that AFD is effective as an independent prognostic model that has the predictive power to identify high-risk groups of patients with LUAD. In addition, these results may indicate a more fundamental role in AFD efficacy in early LUAD detection and accurate survival prediction. However, this study has limitations. First, the number of samples was small, and this limitation could be avoided by conducting a study with a large number of patients. AFD could be applied to measure the effectiveness of medicines by measuring the patient’s response to the treatment used by studying those who used certain treatments. In addition, as a prognostic model, AFD can be applied in further cancer research to verify it in different types of cancer.

## Conclusion

In conclusion, we developed a new prognostic analytical model by developing a new algorithm to calculate the allele frequency deviation (AFD) which characterized by effectiveness predictive performance to predict the survival of LUAD patients. Furthermore, AFD is an independent prognostic tool for predicting survival in patients with LUAD. The study results provided evidence of the possibility of using the AFD in the early diagnosis of patients with LUAD and therefore it may be possible to use AFD in clinical application as a new prognostic tool to predict the patient's outcomes and contribute to follow-up monitoring and help clinicians make effective decisions regarding the potential individual treatment of LUAD patients, which improves their survival. Despite these findings, the model needs further investigation and application in other types of cancers.

## Supplementary Information

**Additional file 1: Table S1.** Source data of clinical information for patients with lung adenocarcinoma (LUAD).

## Data Availability

The raw data used and/or analysed during the current study could be obtained from the European Genome-phenome Archive (EGA) with the accession code EGAS00001004006 (https://ega-archive.org/studies/EGAS00001004006). Source data underlying all figures are provided as an Additional file 1: Table S1.

## Abbreviations

AUC: Area Under Curve
bTMB: Blood Tumor Mutation Burden
CI: Confidence Interval
HR: Hazard Ratio
K-M: Kaplan–Meier
LUAD: Lung Adenocarcinoma
LUSC: Lung Squamous Cell Carcinoma
NSCLC: Non-Small Lung Cancer
OS: Overall Survival
